# What is new in tuberculosis?

**DOI:** 10.1016/j.clinme.2026.100634

**Published:** 2026-08-10

**Authors:** Ye Myat, Maddalena Cerrone, Onn Min Kon

**Affiliations:** a National Heart and Lung Institute, Imperial College London, London, UK; b Department of Respiratory Medicine, Imperial College Healthcare NHS Trust, London, UK; c Department of Infectious Diseases, Imperial College Healthcare NHS Trust, London, UK; d Department of Infectious Diseases, Imperial College London, London, UK

**Keywords:** Tuberculosis, QFT-Plus, Xpert MTB/XDR, WGS, tNGS, Pretomanid

## Abstract

Tuberculosis (TB) is the deadliest leading infectious disease globally. Approximately 10.7 million people worldwide were estimated to have developed TB in 2024. TB rates have increased in the UK over the last three years. Vigilance in acute care settings and across medical subspecialties is essential to ensure early recognition and control of TB particularly given the significant proportion of extrapulmonary presentations. Advances in diagnostic tools have significantly improved detection, with rapid PCR now becoming the standard for timely case identification. Whole genome sequencing (WGS) is now routinely in place to identify drug resistance, track transmission and guide public health responses. In parallel, treatment strategies are evolving, with shorter and more effective regimens available for latent TB, active disease, and multidrug-resistant TB. This review provides an overview of the epidemiology, clinical presentation, diagnosis, and management of TB, with a focus on recent advances relevant to clinical practice in the UK.

## Highlights

• The incidence of tuberculosis (TB) is rising in the UK.
• Xpert MTB/RIF Ultra shows high specificity when used on non-respiratory specimens.
• Whole genome sequencing (WGS) and targeted next-generation sequencing (tNGS) are becoming increasingly adopted globally.
• PET-CT may be particularly useful in the management of complex TB cases.
• WHO now recommends shorter, all-oral regimen for drug-resistant TB (DR-TB).

## Graphical Abstract

**Figure:**
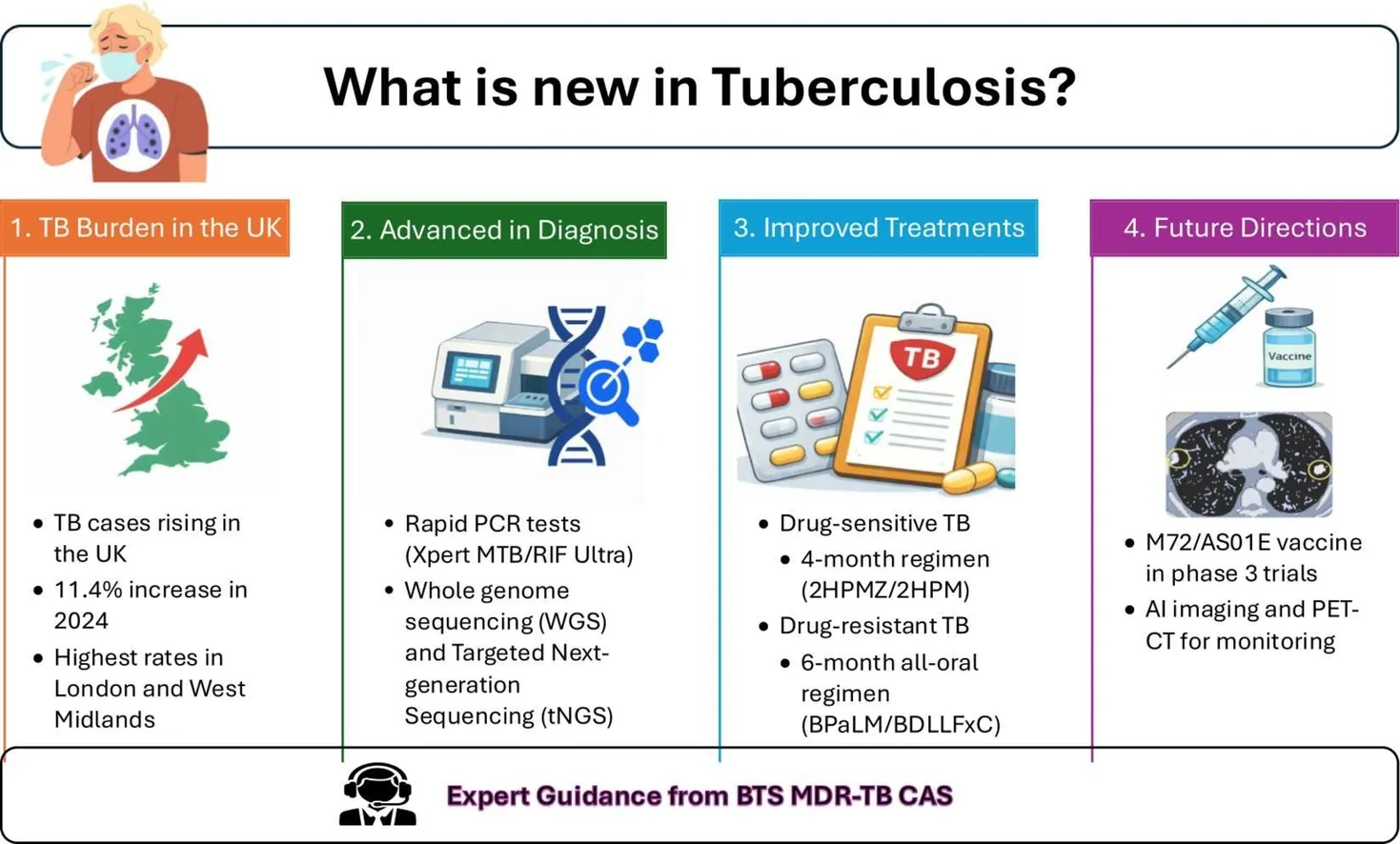

## Introduction

Tuberculosis (TB) is an infectious disease caused by *Mycobacterium tuberculosis* (MTB).^1^ It is characterised by the formation of granulomatous lesions (tubercles) in various organs, most commonly the lungs. Following exposure, MTB infection may remain latent for prolonged periods under immune control, or progress to active disease. Globally, TB remains a major public health challenge, with approximately 10.7 million new cases reported each year.^2^ Around a quarter of the world’s population is estimated to have latent TB infection (LTBI) in which individuals are infected with MTB but do not have active disease or transmit infection, but remain at risk of developing active disease, particularly in the presence of immunosuppression or other risk factors.^2^ The aim of this review is to summarise current evidence on the epidemiology, clinical manifestations, diagnosis, and treatment of TB, including recent developments in molecular diagnostics, management of drug-resistant cases, and implications for clinical practice in the UK.

## Tuberculosis in the UK

The socioeconomic conditions associated with the Industrial Revolution, including widespread poverty, rapid urbanisation, and poor living environments, created ideal conditions for TB transmission, with the disease accounting for approximately one in four deaths by the early nineteenth century.^3^ TB burden declined by an average of 3.3% annually between 1913 and 1940 following the introduction of effective chemotherapy in the late 1950s.^3^ Between 1965 and 1971, TB notifications in England and Wales decreased by 43% among those born in the British Isles, while increasing by 68% among those born in India, Pakistan, and New Commonwealth countries in Africa.^4^

Despite these long-term declines, TB remains a public health concern in the UK. In 2024, the UK remained a low-incidence TB country, with a notification rate of 8.6 per 100,000 population, below the World Health Organization (WHO) threshold of 10 per 100,000.^5^ However, compared with 2023, TB notification rates increased by 11.4%. Increases in TB notification rates were observed across all UK nations, except for Scotland, which had experienced a substantial rise in the preceding year.^5^ The highest TB notification rate in England was observed in London (20.6 per 100,000 population), followed by the West Midlands (11.5 per 100,000 population).^6^ Incidence remains substantially higher in specific areas, with Leicester and Newham in London reporting three-year average notification rates exceeding 40 per 100,000 population in 2024.^5^

These recent increases raise concern that England may exceed the WHO low-incidence threshold in the near future.^7^ This is important because it may signal a reversal of progress towards TB elimination in England and highlights the need to strengthen prevention strategies, including LTBI screening and treatment. People born outside the UK accounted for 81.9% (4,490 of 5,484) of TB notifications in 2024, compared with 80.0% in 2023, representing an increase of more than 8% since 2020. This higher burden largely reflects migration from high-TB-incidence countries, with one-third of TB notifications among people born outside the UK occurring in individuals born in India, followed by Pakistan, Nigeria, and Romania.^6^ In 2024, TB diagnoses among people born outside the UK within five years of arrival increased by 23.2%, with diagnoses within one to two years of entry rising by 42.4%. This trend is likely driven by increased migration from high-TB-incidence countries and the lasting impact of COVID-19-related disruptions to TB services.^6^ To address the high burden of TB among people born outside the UK, the National TB Action Plan (2021–2026) includes post-entry LTBI testing and treatment, alongside pre-entry screening for active pulmonary TB, with the aim of reducing active TB notifications among recent migrants by 5% annually.^8^ TB notification rates also increased among UK-born individuals, rising by 5.0% in 2024.^6^

## Risk factors for tuberculosis

The risk of developing TB is increased with HIV infection, malnutrition, and young adults; risk is also elevated with diabetes, immunosuppressive therapies, chronic liver or renal disease, smoking, and exposure to indoor air pollution.^6^ In recent years, the introduction of novel biological therapies targeting the immune system, for instance to treat rheumatological or dermatological conditions, has heightened the need to assess patients for TB reactivation risk. Tumour necrosis factor-α (TNF-α) plays a critical role in maintaining granulomas that contain latent MTB infection, and inhibition of this pathway can lead to reactivation and progression to active disease.^9^ Consequently, screening and prophylaxis are often recommended, and local guideline should be followed. The risk of TB varies substantially across biologic therapies (Table 1), with the highest relative risks reported for adalimumab and infliximab (29.3 and 18.6, respectively), whereas newer TNF-α inhibitors and biologic agents appear to be associated with lower TB risk.^10^

**Table 1:** Relative risk of TB reactivation associated with commonly used biologic and targeted immunomodulatory therapies.

Drug class	Examples	Relative TB risk
Anti-TNF monoclonal antibodies	Infliximab, Adalimumab	High
TNF receptor fusion protein	Etanercept	Moderate
Anti-IL-6	Tocilizumab	Low–moderate
Anti-IL-12/23	Ustekinumab	Low
Anti-integrin	Vedolizumab	Low
Anti-IL-12/23	Ustekinumab	Low
Anti-CD20	Rituximab	Low

Importantly, TB is also more common among dialysis patients and kidney transplant recipients, particularly during the first year of dialysis.^11^ Additionally, Lange *et al*. demonstrated that solid organ transplant recipients have an increased risk of TB even in low- to medium-incidence countries in Europe.^12^

## Advances in TB diagnosis

**Fig. 1:**
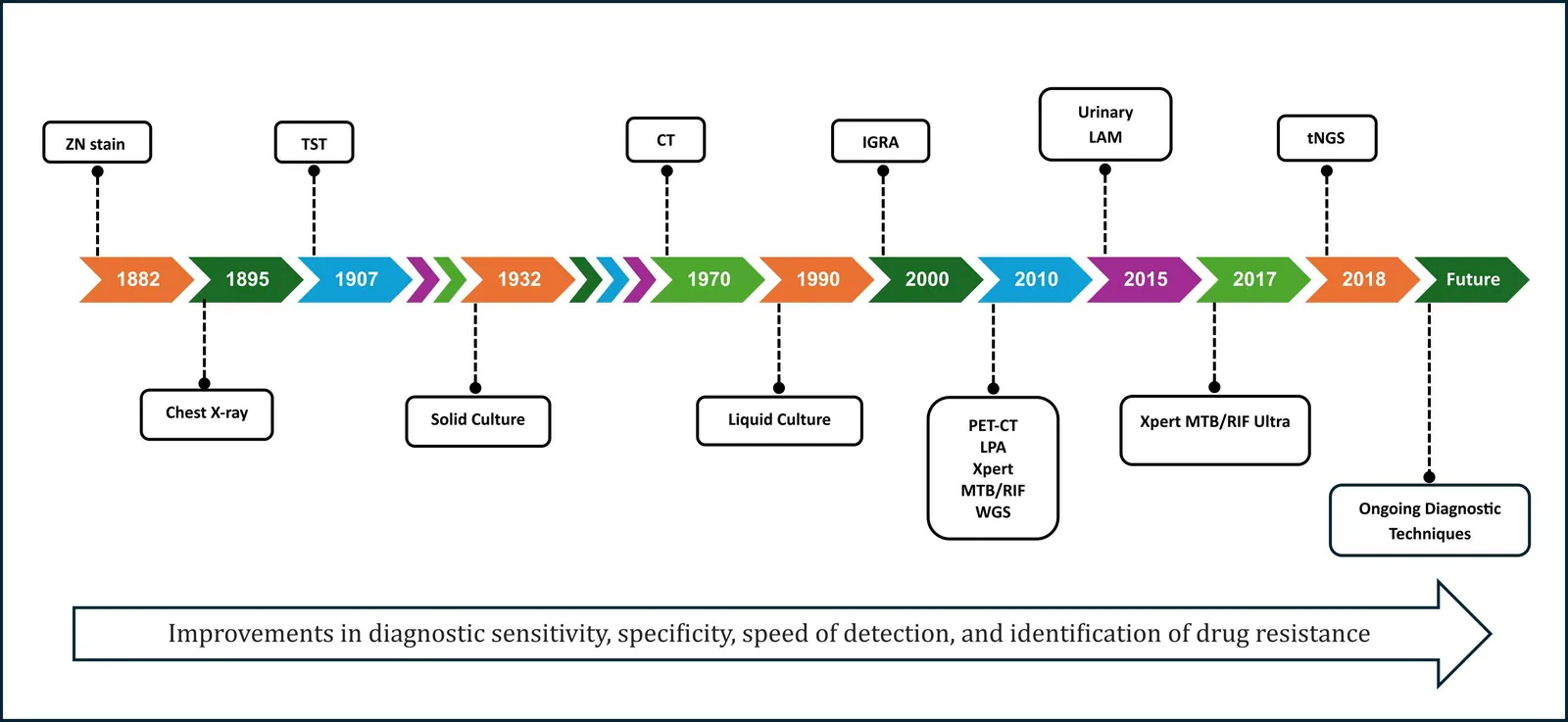
Spectrum of established and emerging diagnostic investigations for tuberculosis, from conventional microscopy and culture to molecular, imaging, and sequencing-based technologies.

In recent years, several key developments have transformed the management of TB.^13^ Diagnostic pathways have improved, with expanded LTBI screening enabling earlier identification of individuals at risk of progression. Rapid molecular testing using PCR has become increasingly integrated into clinical practice, allowing faster confirmation of TB and early detection of drug resistance^14^ (Fig. 1). The 2023 UN High-Level Meeting on Tuberculosis target calls for all individuals diagnosed with TB to undergo initial testing with a WHO-recommended rapid diagnostic test (WRD), including PCR testing. Although global coverage increased from 48% in 2023 to 54% in 2024, progress remains well below the target of universal access by 2027.^2^ In England, the current 2016 NICE guidance recommends rapid molecular testing only in selected clinical scenarios, including suspected pulmonary or laryngeal tuberculosis in people living with HIV, when rapid identification of mycobacterial species would influence clinical management or large-scale contact tracing, in children younger than 15 years, and in selected cases of extrapulmonary TB where the result would alter management.^15^ Although the guideline is due for review, the NICE Prioritisation Board decided in January 2025 not to update these recommendations.^16^ In this context, although the use of PCR testing for TB diagnosis has increased in England, preliminary unpublished data from the UK Health Security Agency (UKHSA) National Laboratory Audit (2023) suggest that it is not yet routinely used as the initial diagnostic test across all laboratories, highlighting the need for updated guidance to support its universal implementation as a first-line diagnostic test.

The Whole genome sequencing (WGS) is now increasingly used to support surveillance, guide public health interventions, as well as for resistance prediction for first- and second-line drugs.

### Rapid molecular tests

Traditional diagnostic methods such as smear microscopy have limited sensitivity, particularly in paucibacillary disease, while culture, although highly sensitive, may take several weeks to provide results. Rapid molecular tests overcome these limitations by detecting specific DNA sequences of MTB and genetic mutations associated with drug resistance directly from clinical specimens, enabling earlier diagnosis and treatment. Examples include the HAIN line probe assay (LPA), Xpert MTB/RIF Ultra (Cepheid) and Truenat MTB Plus (Molbio).^14^

Xpert MTB/RIF Ultra is widely used in UK laboratories for the diagnosis of TB and rifampicin resistance. This test and has high diagnostic accuracy, with an overall sensitivity of 91% and specificity of 95% for pulmonary TB compared with culture. Performance remains high in key patient groups, including people living with HIV (87.7% sensitivity), individuals with smear-negative pulmonary TB (80.7%), and those with a history of TB (84.8%), while maintaining specificity above 86% across all groups.^17^

When used on non-respiratory specimen, Xpert MTB/RIF Ultra demonstrates high specificity (>98%), however sensitivity varies by extrapulmonary specimen type, ranging approximately from 50% to over 80%, with higher sensitivities reported for lymph node tissue, cerebrospinal fluid, and bone or joint samples.^18^

Xpert MTB/RIF Ultra also shows excellent accuracy for detecting rifampicin resistance, with a sensitivity of 95.8% and specificity of 98.3% compared with culture-based drug susceptibility testing.^17^

More recently, the Xpert MTB/XDR assay is a PCR test developed to detect resistance to second-line anti-tuberculosis drugs, including isoniazid, fluoroquinolones and injectable aminoglycosides. It is intended for use as a follow-on test after an initial molecular assay has identified rifampicin resistance. Although the assay is generally not routinely available in local laboratories across England, testing can be accessed through the National Mycobacterial Reference Laboratory.

With sensitivities >94% for isoniazid and >91% for fluoroquinolone resistance, and specificity >90%, the test performs well overall. Although sensitivity for second-line injectable resistance is lower, high specificity supports its use as a rapid molecular tool to guide treatment of drug-resistant TB.^19^, ^20^, ^21^

Amongst other technologies already available within some laboratories in the UK, the recently introduced FluoroType® Mycobacteria assay is a molecular diagnostic platform analogous to the established GenoType system, enabling identification of the MTB complex and up to 32 clinically relevant non-tuberculous mycobacterial species from culture isolates.^22^ Of note, results are generated more rapidly than with whole-genome sequencing, facilitating earlier treatment decisions.

### Advanced molecular tests: whole genome sequencing (WGS) and targeted next-generation sequencing (tNGS)

Unlike targeted PCR-based assays, WGS and tNGS provide substantially more comprehensive genetic information and can identify a broader range of resistance-conferring mutations. WGS and tNGS platforms are being increasingly adopted worldwide for TB diagnostics and surveillance.^23^ WGS enables rapid and comprehensive analysis of MTB, with results available within approximately five working days of a culture-positive sample, depending on the laboratory set-up and bioinformatic pipeline.^24^ Advances in sequencing technology now allow accurate sequencing of the MTB genome directly from uncultured sputum specimens, bypassing prolonged culture.^25^ Nevertheless, in practice, particularly in settings such as the UK where bacillary load is generally not very high, WGS is routinely performed by the reference laboratories on positive cultures to enhance sensitivity.^26^ Importantly, WGS, through comprehensive analysis of the mycobacterial genome, provides high-resolution strain differentiation for lineage classification and evaluation of genetic relatedness.^26^ It further facilitates genotypic resistance prediction to first-line, second-line, and newer MDR-TB agents, and is extensively utilised in surveillance and public health interventions, including outbreak investigation and transmission mapping by linking phylogenetically related isolates.^27^ Moreover, the bioinformatic detection of drug resistance is based on the WHO mutation catalogue, which is continuously updated and, in many cases, exclusion of resistance still requires a traditional *in vitro* sensitivity test.^27^

tNGS approaches such as Deeplex Myc-TB can be performed directly on clinical samples, particularly smear-positive samples.^28^ Unlike WGS, which analyses the entire mycobacterial genome, tNGS targets selected genomic regions associated with drug resistance, enabling rapid resistance profiling directly from clinical specimens. tNGS can also detect resistance to rifampicin and other antibiotics, providing a rapid molecular alternative to culture-based phenotypic drug susceptibility testing,^14^ although phenotypic confirmation may still be required in selected cases. tNGS is not yet been widely implemented in many countries, including the UK.

### Urinary antigen test

Lipoarabinomannan (LAM), a component of the lipid-rich mycobacterial cell wall, is a highly specific antigen that is excreted in urine and can be used as a diagnostic marker.^29^ The Alere Determine TB LAM antigen assay (AlereLAM) is currently the only WHO-recommended test for detecting urinary LAM and is indicated for patients with HIV-associated immunosuppression, particularly those with disseminated TB.^14^ Despite their routine use in countries with a higher burden of HIV, these tests have not yet been incorporated into standard practice in the UK where advanced HIV-associated disseminated TB is an uncommon presentation.

### Radiological imaging

The integration of ^18^F-fluorodeoxyglucose positron emission tomography (^18^F-FDG PET) with computed tomography (CT) imaging, despite its high cost, is emerging as a valuable tool in TB management^13^ although its current use is largely limited to research studies and selected specialised centres. In the context of multidrug-resistant and extensively drug-resistant TB, the use of PET-CT may be particularly valuable in guiding the management of complex cases when ensuring disease clearance is crucial or where activity may prompt additional sampling of equivocal lesions.^13^ Evidence from cohort studies shows that reduced PET glycolytic activity correlates with effective treatment and can help predict outcomes.^30^

Finally, AI-based volumetric CT analysis can determine the activity status of pulmonary lesions, including tuberculomas, improving diagnostic accuracy.^31^ However, further validation studies are required.

### Tests for latent TB infection

IGRAs are blood tests that quantify the release of interferon gamma by T cells upon *in vitro* stimulation with antigens specifics for MTb.^32^ A IGRA-positive result indicates TB infection but does not distinguish between latent infection and active disease, which is an important diagnostic limitation.^33^ Similarly, a positive tuberculin skin test (TST) may reflect TB infection or prior BCG vaccination and is also not diagnostic of active disease. Only a small proportion of those infected progress to active TB, highlighting the need for clinical, microbiological, and radiological assessment to confirm disease.

Amongst the IGRAs assays commercially available, there are two widely used in the UK: QuantiFERON-TB Gold Plus (QFT-Plus) and T-SPOT.TB assay. QFT-Plus is a newer generation IGRA developed to replace the QuantiFERON-TB Gold In-Tube (QFT-GIT) assay.^34^ QFT-Plus quantifies interferon-γ (IFN-γ) concentration using an enzyme-linked immunosorbent assay (ELISA) performed on a defined volume of whole blood and includes separate antigen tubes designed to stimulate CD4⁺ T-cell responses (TB1) and combined CD4⁺/CD8⁺ T-cell responses (TB2).^34^ IGRAs offer several advantages over the TST, including a single patient visit, results available within 24 h, reliability in individuals with prior BCG vaccination, and the absence of reader bias.^35^ However, immunosuppressed patients may have false-negative^35^ or indeterminate results.^36^ The latter occurs more frequently because the positive control (mitogen) fails to generate an adequate IFN-γ response, but they may also result from failure of the negative control tube. IGRAs are not recommended for children under five years of age. In addition, IGRA tests are not suitable for monitoring treatment response.^35^

### *Mycobacterium tuberculosis* antigen–based skin tests (TBSTs)

TBSTs are newer intradermal tests, not yet routinely used in the UK. These include Diaskintest, ESAT-6/CFP-10–based tests (C-TST), C-Tb (Cy-Tb), and DPPD.^37^ Unlike the traditional TST, TBSTs use *M. tuberculosis*-specific antigens, which may reduce cross-reactivity associated with BCG vaccination and some non-tuberculous mycobacteria.^38^ A systematic review has shown that the diagnostic accuracy of TBSTs for latent TB infection is comparable to that of IGRAs.^39^ In addition, the safety profile of TBSTs appears similar to that of the traditional TST.^37^

## Treatment and vaccine pipeline

### Shorter regimen for drug-sensitive TB (DS-TB)

According to the WHO, the current recommended treatment for DS-TB is a six-month regimen^40^:

• Intensive phase (two months): Isoniazid (H), Rifampicin (R), Pyrazinamide (Z), and Ethambutol (E)
• Continuation phase (four months): Isoniazid (H) and Rifampicin (R)

This 2HRZE/4HR regimen is recommended for adults, adolescents, and children with drug-sensitive pulmonary TB.^40^

In recent years, efforts have focused on evaluating whether treatment regimens shorter than six months are sufficient to cure TB, particularly in uncomplicated cases. A large international, multicentre, randomised phase 3 non-inferiority trial (Study 31/A5349) evaluated whether a four-month rifapentine-based regimen, with or without moxifloxacin, could replace the standard six-month treatment for drug-sensitive pulmonary tuberculosis.^41^ A total of 2,343 participants aged ≥12 years were enroled, including people living with HIV. The rifapentine–moxifloxacin regimen was shown to be non-inferior to the standard six-month regimen, with unfavourable outcomes occurring in 15.5% of participants compared with 14.6% in the control group. The primary endpoint was survival free of TB at 12 months. Follow-up lasted 18 months. These findings support the use of a shorter, four-month rifapentine-based regimen for selected patients with drug-sensitive TB.^41^ However, the current difficulties with being able to access rifapentine given the lack of licensing in western Europe has meant this region is unable to routinely use this shorter regimen for its patients.^42^

The SHINE trial has similarly demonstrated that four months of antituberculosis treatment (two months of isoniazid, rifampicin and pyrazinamide with or without ethambutol followed by two months of isoniazid and rifampicin) was non-inferior to six months of therapy in children with drug-susceptible, non-severe, smear-negative tuberculosis.^43^

### Shorter all oral regimen for drug-resistant TB (DR-TB)

**Fig. 2:**
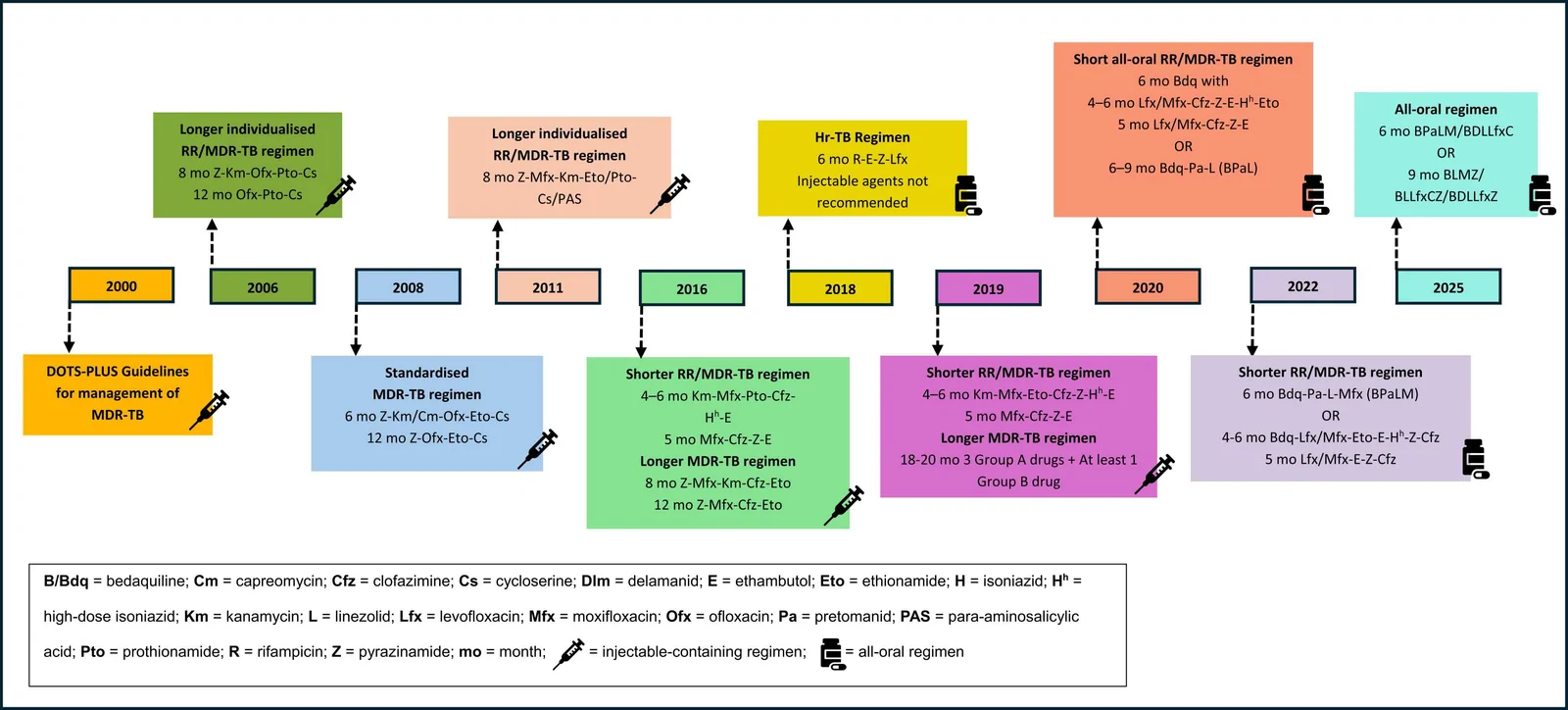
Timeline showing the evolution of WHO-recommended regimens for drug-resistant tuberculosis from 2000 to 2025. Treatment progressed from long, injectable-based DOTS-Plus regimens to shorter standardised regimens and, subsequently, to predominantly all-oral, bedaquiline-based combinations.

Recent trials have reshaped the management of MDR-TB by supporting shorter, all-oral regimens (Fig. 2). Historically, the treatment of drug-resistant tuberculosis involved prolonged regimens lasting approximately 18–24 months with multiple less effective, more toxic second-line drugs, contributing to poor tolerability and unfavourable outcomes^44^

The STREAM Stage 2 study showed superior efficacy and reduced hearing loss with bedaquiline-containing regimens compared with injectable-based therapy.^45^ The TB-PRACTECAL trial further demonstrated that a six-month all-oral regimen was more effective and safer than standard treatment for rifampicin-resistant TB.^46^ Similarly, the Nix-TB and ZeNix studies demonstrated high cure rates using bedaquiline, pretomanid, and linezolid, with ZeNix optimising linezolid dosing to improve safety.^47^, ^48^ The NEXT study further confirmed the effectiveness of a six-month all-oral regimen.^49^ In addition, the BEAT-TB regimen, consisting of bedaquiline, delamanid, and linezolid combined with levofloxacin and/or clofazimine for six months, was found to be non-inferior to the standard-of-care treatment for RR-TB in South Africa.^50^ Moreover, the endTB trial indicated that three shorter, nine month all-oral regimens – BLMZ (bedaquiline, linezolid, moxifloxacin, and pyrazinamide), BCLLfxZ (bedaquiline, clofazimine, linezolid, levofloxacin, and pyrazinamide), and BDLLfxZ (bedaquiline, delamanid, linezolid, levofloxacin, and pyrazinamide) were noninferior to standard therapy for rifampicin-resistant TB, achieving favourable outcomes in more than 85% of participants.^51^ Together, these findings underpin current WHO recommendations for shorter, safer MDR-TB treatment.

For patients with rifampicin-resistant or multidrug-resistant TB, the preferred option is a six–nine-month all-oral regimen such as BPaLM (bedaquiline, pretomanid, linezolid, and moxifloxacin).^40^ In cases with fluoroquinolone resistance or intolerance, BPaL (bedaquiline, pretomanid, and linezolid) may be used without moxifloxacin. Longer individualised regimens, typically lasting 9–18 months or longer, are reserved for patients who are not eligible for shorter regimens. WHO recommends constructing these regimens using its classification of second-line anti-tuberculosis drugs, prioritising all three Group A drugs (bedaquiline, linezolid and a fluoroquinolone), followed by one or both Group B drugs (clofazimine and cycloserine/terizidone), with Group C agents used only when an effective regimen cannot be achieved using Groups A and B.^40^

Compared with a decade ago, the anti-tuberculosis drug pipeline has strengthened, encompassing both repurposed antibiotic classes alongside novel drug candidates with new mechanisms of action.^52^ However, despite the availability and effectiveness of these treatments, major obstacles persist in achieving durable disease control and global TB eradication including limited vaccine trial sustainability, difficulties in epitope selection, exclusion of pregnant women, uncertainty around trial endpoints, restricted adjuvant and delivery platforms, and a lack of suitable animal models.^53^

In this context, vaccine development has gained renewed importance. The tuberculosis vaccine pipeline is at a pivotal stage, with significant advances in clinical development occurring despite persistent scientific and political challenges.^54^ The M72/AS01E TB vaccine candidate, possibly the most promising under evaluation at the moment, demonstrated approximately 50–54% efficacy against active pulmonary TB in a phase 2b trial of adults with latent *M. tuberculosis* infection, with an acceptable safety profile, and is now being evaluated in a large phase 3 trial.^55^

## British Thoracic Society multidrug-resistant tuberculosis clinical advice service (BTS MDR-TB CAS)

In response to the increasing complexity of DR-TB management and the rapid evolution of diagnostic and therapeutic strategies, the UK established a national clinical advisory framework to support clinicians managing multidrug-resistant disease in January 2018. This built on the longstanding work of the former MDR-TB Forum initially established in 2008, allowed for advice in the management of multidrug-resistant tuberculosis in the UK by providing an online case discussion.

The transition to the BTS MDR-TB CAS formalised and expanded this role, integrating advances in diagnostics, therapeutics, and national policy into a robust advisory framework which, in addition to clinicians, includes reference laboratory microbiologists, public health and pharmacists. In addition to a rapid online response facility, there is a monthly virtual MDT where clinicians can present and discuss cases with the advisers. The service facilitates timely, evidence-based clinical decision-making, promotes consistency of care across the UK, and supports the implementation of evolving WHO-aligned treatment recommendations. In addition, the CAS serves as an important platform for shared learning, capacity building, and surveillance, contributing to improved clinical outcomes and strengthened national expertise in the management of drug-resistant tuberculosis.

## CRediT authorship contribution statement

**Ye Myat:** Writing – original draft. **Onn Min Kon:** Writing – review & editing, Conceptualization. **Maddalena Cerrone:** Writing – review & editing.

## Funding

OMK is supported by the 10.13039/501100013342NIHR Imperial Biomedical Research Centre (BRC).

## Declaration of competing interest

The authors declare that they have no known competing financial interests or personal relationships that could have appeared to influence the work reported in this paper.

## References

[1] Alsayed S.S.R., Gunosewoyo H. (2023). Tuberculosis: pathogenesis, current treatment regimens and new drug targets. Int J Mol Sci.

[2] World Health Organization (2025). Global Tuberculosis Report 2025.

[3] Glaziou P., Floyd K., Raviglione M. (2018). Trends in tuberculosis in the UK. Thorax.

[4] Research Committee of the British Thoracic and Tuberculosis Association (1973). A tuberculosis survey in England and Wales 1971; the influence of immigration and country of birth upon notifications: a report from the Research Committee of the British Thoracic and Tuberculosis Association. Tubercle.

[5] UK Health Security Agency (2025). Reports of Cases of TB to UK Enhanced Tuberculosis Surveillance Systems, England: 2000–2024 Report.

[6] Cox S.E., Presslie O., Khan A.H. (2025).

[7] Wise J. (2024). Tuberculosis: health chiefs issue warning over rise in cases. BMJ.

[8] UK Health Security Agency, NHS England (2023). Tuberculosis (TB): Action Plan for England, 2021–2026.

[9] Aliravci I.D., Mutlu P., Oymak S., Guney U.I., Keskin O. (2025). Risk of latent tuberculosis infection reactivation in patients treated with tumor necrosis factor antagonists: a five-year retrospective study. Trop Med Infect Dis.

[10] Dobler C.C. (2016). Biologic agents and tuberculosis. Microbiol Spectr.

[11] Ali M., Dosani D., Corbett R. (2022). Diagnosis of tuberculosis in dialysis and kidney transplant patients. Hemodial Int.

[12] Lange B., Brehm T.T., Arend S.M. (2026). Tuberculosis incidence in solid organ transplant recipients in Europe: a multicenter TBnet cohort study. J Infect.

[13] Shrivas A., Singh S. (2025). Tuberculosis diagnosis and management: recent advances. J Glob Infect Dis.

[14] World Health Organization (2024). WHO Consolidated Guidelines on Tuberculosis. Module 3: Diagnosis – Rapid Diagnostics for Tuberculosis Detection.

[15] National Institute for Health and Care Excellence (2016).

[16] National Institute for Health and Care Excellence (2025).

[17] Horne D.J., Zifodya J.S., Shapiro A.E. (2025). Xpert MTB/RIF Ultra assay for pulmonary tuberculosis and rifampicin resistance in adults and adolescents. Cochrane Database Syst Rev.

[18] Park M., Kon O.M. (2021). Use of Xpert MTB/RIF and Xpert Ultra in extrapulmonary tuberculosis. Expert Rev Anti Infect Ther.

[19] Zhang R., Bao X., Bao F. (2025). Evaluation of the Xpert MTB/XDR test for detection of isoniazid, fluoroquinolones, and second-line injectable drugs resistance to Mycobacterium tuberculosis-Anhui Province, China. PLoS One.

[20] Centner C.M., Munir R., Tagliani E. (2024). Reflex Xpert MTB/XDR testing of residual rifampicin-resistant specimens: a clinical laboratory-based diagnostic accuracy and feasibility study in South Africa. Open Forum Infect Dis.

[21] Pillay S., Steingart K.R., Davies G.R. (2022). Xpert MTB/XDR for detection of pulmonary tuberculosis and resistance to isoniazid, fluoroquinolones, ethionamide, and amikacin. Cochrane Database Syst Rev.

[22] Luukinen B., Antikainen J., Aittoniemi J., Miikkulainen-Lahti T., Hyyrylainen H.L., Patari-Sampo A. (2024). Evaluation of the fluorotype Mycobacteria assay for identification of mycobacteria. J Clin Microbiol.

[23] Ghodousi A., Cannas A., Tagliani E. (2025). Comprehensive whole genome sequencing dataset of Mycobacterium tuberculosis strains collected across Italy. Sci Data.

[24] Doyle R.M., Burgess C., Williams R. (2018). Direct whole-genome sequencing of sputum accurately identifies drug-resistant Mycobacterium tuberculosis faster than MGIT culture sequencing. J Clin Microbiol.

[25] Nimmo C., Shaw L.P., Doyle R. (2019). Whole genome sequencing Mycobacterium tuberculosis directly from sputum identifies more genetic diversity than sequencing from culture. BMC Genom.

[26] UK Health Security Agency (2022).

[27] World Health Organization (2023). Catalogue of Mutations in Mycobacterium tuberculosis Complex and their Association With Drug Resistance.

[28] Schwab T.C., Perrig L., Goller P.C. (2024). Targeted next-generation sequencing to diagnose drug-resistant tuberculosis: a systematic review and meta-analysis. Lancet Infect Dis.

[29] Li Y., Ru Z., Wei H. (2024). Improving the diagnosis of active tuberculosis: a novel approach using magnetic particle-based chemiluminescence LAM assay. BMC Pulm Med.

[30] Heyckendorf J., Georghiou S.B., Frahm N. (2022). Tuberculosis treatment monitoring and outcome measures: new interest and new strategies. Clin Microbiol Rev.

[31] Wei G., Qiu X., Xu Y., Zhan M., Qian Z. (2024). Study on artificial intelligence to judge the activity of tuberculomas. J Radiat Res Appl Sci.

[32] Rockwood N., du Bruyn E., Morris T., Wilkinson R.J. (2016). Assessment of treatment response in tuberculosis. Expert Rev Respir Med.

[33] Pai M., Behr M.A., Dowdy D. (2016). Tuberculosis. Nat Rev Dis Primers.

[34] Rudeeaneksin J., Srisungngam S., Klayut W., Bunchoo S., Bhakdeenuan P., Phetsuksiri B. (2023). QuantiFERON-TB Gold Plus and QuantiFERON-TB Gold In-tube assays for detecting latent tuberculosis infection in Thai healthcare workers. Rev Inst Med Trop Sao Paulo.

[35] Pai M., Denkinger C.M., Kik S.V. (2014). Gamma interferon release assays for detection of Mycobacterium tuberculosis infection. Clin Microbiol Rev.

[36] Zhou G., Luo Q., Luo S. (2023). Indeterminate results of interferon gamma release assays in the screening of latent tuberculosis infection: a systematic review and meta-analysis. Front Immunol.

[37] Hamada Y., Kontsevaya I., Surkova E. (2023). A systematic review on the safety of Mycobacterium tuberculosis-specific antigen-based skin tests for tuberculosis infection compared with tuberculin skin tests. Open Forum Infect Dis.

[38] van Pinxteren L.A., Ravn P., Agger E.M., Pollock J., Andersen P. (2000). Diagnosis of tuberculosis based on the two specific antigens ESAT-6 and CFP10. Clin Diagn Lab Immunol.

[39] Krutikov M., Faust L., Nikolayevskyy V. (2022). The diagnostic performance of novel skin-based in-vivo tests for tuberculosis infection compared with purified protein derivative tuberculin skin tests and blood-based in vitro interferon-gamma release assays: a systematic review and meta-analysis. Lancet Infect Dis.

[40] World Health Organization (2025). WHO Consolidated Guidelines on Tuberculosis. Module 4: Treatment and Care.

[41] Dorman S.E., Nahid P., Kurbatova E.V. (2021). Four-month rifapentine regimens with or without moxifloxacin for tuberculosis. N Engl J Med.

[42] Guglielmetti L., Gunther G., Leu C. (2022). Rifapentine access in Europe: growing concerns over key tuberculosis treatment component. Eur Respir J.

[43] Turkova A., Wills G.H., Wobudeya E. (2022). Shorter treatment for nonsevere tuberculosis in African and Indian children. N Engl J Med.

[44] Jang J.G., Chung J.H. (2020). Diagnosis and treatment of multidrug-resistant tuberculosis. Yeungnam Univ J Med.

[45] Goodall R.L., Meredith S.K., Nunn A.J. (2022). Evaluation of two short standardised regimens for the treatment of rifampicin-resistant tuberculosis (STREAM stage 2): an open-label, multicentre, randomised, non-inferiority trial. Lancet.

[46] Nyang'wa B.T., Berry C., Kazounis E. (2022). A 24-week, all-oral regimen for rifampin-resistant tuberculosis. N Engl J Med.

[47] Conradie F., Bagdasaryan T.R., Borisov S. (2022). Bedaquiline-pretomanid-linezolid regimens for drug-resistant tuberculosis. N Engl J Med.

[48] Conradie F., Diacon A.H., Ngubane N. (2020). Treatment of highly drug-resistant pulmonary tuberculosis. N Engl J Med.

[49] Esmail A., Oelofse S., Lombard C. (2022). An all-oral 6-month regimen for multidrug-resistant tuberculosis: a multicenter, randomized controlled clinical trial (the NExT study). Am J Respir Crit Care Med.

[50] Conradie F., Badat T., Poswa A. (2025). BEAT tuberculosis: a randomized controlled trial of a 6-month strategy for rifampicin-resistant tuberculosis. medRxiv.

[51] Guglielmetti L., Khan U., Velasquez G.E. (2025). Oral regimens for rifampin-resistant, fluoroquinolone-susceptible tuberculosis. N Engl J Med.

[52] Dartois V.A., Rubin E.J. (2022). Anti-tuberculosis treatment strategies and drug development: challenges and priorities. Nat Rev Microbiol.

[53] Zhuang L., Ye Z., Li L., Yang L., Gong W. (2023). Next-generation TB vaccines: progress, challenges, and prospects. Vaccines.

[54] Konjengbam B.D., Meitei H.N., Pandey A., Haobam R. (2025). Goals and strategies in vaccine development against tuberculosis. Mol Immunol.

[55] Tait D.R., Hatherill M., Van Der Meeren O. (2019). Final analysis of a trial of M72/AS01(E) vaccine to prevent tuberculosis. N Engl J Med.

